# Genome-wide analysis reveals a role for TDG in estrogen receptor-mediated enhancer RNA transcription and 3-dimensional reorganization

**DOI:** 10.1186/s13072-018-0176-2

**Published:** 2018-01-29

**Authors:** Bart Kolendowski, Haider Hassan, Milica Krstic, Majdina Isovic, Gobi Thillainadesan, Ann F. Chambers, Alan B. Tuck, Joseph Torchia

**Affiliations:** 1 Department of Biochemistry, Western University, London, ON Canada; 2 Department of Oncology, Western University, London, ON Canada; 3 Department of Pathology, Western University, London, ON Canada; 4 The London Regional Cancer Program and the Lawson Health Research Institute, Western University, London, ON Canada; 5 Present Address: Cancer Research Laboratories, London Regional Cancer Program, London, ON N6A 4L6 Canada

**Keywords:** Cancer, Thymine DNA glycosylase, Estrogen receptor, eRNA, Estrogen signaling

## Abstract

**Background:**

The estrogen receptor (ER) is a ligand-dependant transcription factor expressed in many breast cancers and is the target of many endocrine-based cancer therapies. Genome-wide studies have shown that the ER binds to gene-specific enhancer regions in response to β-estradiol (E2) which undergo transcription producing noncoding enhancer RNA (eRNA). While eRNAs are important for transcriptional activation of neighboring genes, the mechanism remains poorly understood.

**Results:**

Using ChIP-Seq we generate a global profile of thymine DNA glycosylase (TDG), an ER coactivator that plays an essential role in DNA demethylation, in response to E2 in the MCF7 breast cancer cell line. Remarkably, we found that in response to E2 TDG localized to enhancers which also recruit ERα, RNA Pol II and other coregulators and which are marked by histone modifications indicative of active enhancers. Importantly, depletion of TDG inhibits E2-mediated transcription of eRNAs and transcription of ER-target genes. Functionally, we find that TDG both sensitizes MCF7 cells to tamoxifen-mediated cytostasis and increases migration and invasion of MCF7 cells.

**Conclusions:**

Taken together we find that TDG plays a central role in mediating transcription at a subset of enhancers and governs how MCF7 cells respond to both estrogenic and anti-estrogenic compounds and may be an effective therapeutic target.

**Electronic supplementary material:**

The online version of this article (10.1186/s13072-018-0176-2) contains supplementary material, which is available to authorized users.

## Background

Steroid hormones such as 17β-estradiol (E2) coordinate complex gene programs and exert profound effects on cell growth, development and homeostasis [1]. E2 mediates its biological effects by binding to, and activating, the estrogen receptor (ERα and ERβ). The ERs are members of the nuclear hormone receptor superfamily which function as ligand-activated transcription factors. In the classical mechanism of hormone action, E2 binding induces receptor dimerization which facilitates binding to genomic DNA at specific sequences in the regulatory region of ER responsive genes called “estrogen response elements” (ERE). Importantly, ligand-bound ER undergoes a conformational change that facilitates the recruitment of coactivator proteins that coordinate specific transcriptional responses.

Genome-wide studies using ChIP-based technologies have shown that the majority of ERα binding sites in breast cancer cells are found distally from gene promoters, and a significant component is found within gene-specific “enhancer” regions in response to the E2 [2]. Enhancers are essential regulatory regions found in noncoding regions that control temporal and tissue-specific gene expression. Furthermore, given that less than 2% of the mammalian genome accounts for protein-coding genes, an increasing number of mutations and aberrant methylation patterns associated with breast cancer have been found to reside in enhancer regions [3]. In addition to recruiting specific transcription factors enhancers also bind specific coregulators and components of the transcriptional machinery, including RNA polymerase II. Importantly, some enhancers are actively transcribed into long noncoding RNAs known as enhancer RNAs (eRNAs) [4]. While the exact role of eRNAs remains controversial, some eRNAs have been shown to regulate gene expression by causing a 3-dimensional conformational change bringing together the promoter, enhancer and transcriptional machinery into “transcriptional pockets” [4, 5]. It has been shown that E2 rapidly increases eRNA production at many sites of ERα binding and results in the activation of adjacent genes [4]. Although the exact mechanism governing eRNA transcription is unclear, recent evidence suggests that enhancer methylation status may play a role in eRNA production [6].

DNA methylation occurs at the C5 position of cytosine (5mC) and is found primarily in a cytosine–guanine (CpG) context. Genome-wide patterns of CpG methylation are deposited by the DNA methyltransferases (DNMTs) and are important for the establishment of proper chromatin states that are associated with normal development and cellular homeostasis [7]. 5mCs function as targets for methyl-binding domain proteins which can subsequently recruit additional chromatin remodelers and co-repressors [8]. Furthermore, in a promoter context, methylated CpGs can render the site inaccessible to the transcriptional machinery resulting in transcriptional silencing. Interestingly, while the majority of genomic DNA is methylated at CpGs [9], 40–70% of gene promoters contain long stretches of CpG clusters (CpG islands) that are unmethylated based on bisulfite sequencing analysis [10]. This pattern of global hypermethylation and promoter hypo-methylation is present in healthy tissue and in differentiated cell types. Importantly, improper control of the setting and erasure of these marks has been implicated in various pathological phenotypes, such as cancer and abnormal embryogenesis [11, 12].

Whereas the mechanism of DNA methylation is well understood, a unifying mechanism for DNA demethylation has not been unequivocally identified. DNA demethylation may occur passively when newly synthesized DNA strands remain unmethylated during successive rounds of DNA replication, as a result of DNMT1 inhibition. In contrast, active demethylation is a replication-independent process involving the DNA glycosylase Thymine DNA glycosylase (TDG). In one scenario, cytidine deaminases such as activation-induced deaminase (AID) or Apolipoprotein B mRNA editing enzymes (APOBEC 1-4) convert 5mC to thymine, generating a G/T mispair [13]. Excision of mispaired thymine by TDG initiates the base excision repair pathway (BER) which effectively restores unmethylated cytosine. However, this model has been challenged recently because AID/APOBEC members are much less active on 5mC and its derivatives in vitro and in vivo [14]. A more plausible mechanism involves the ten eleven translocation (TET 1-3) enzymes which oxidize 5mC to 5-hydroxymethylcytosine (5hmC). Subsequently, 5hmC is then metabolized further by TETs into 5-formylcytosine (5fC) and 5-carboxylcytosine (5caC) [15]. These oxidized 5mC metabolites, 5fC and 5caC, are recognized and removed by TDG [16, 17]. In addition, an alternative mechanism has been postulated for ER-dependent demethylation in breast cancer cells [18]. ER-dependant transcriptional activation at the TFF1 promoter requires cyclic patterns of methylation and demethylation that is mediated by recruitment of TDG in concert with DNMT3a/b [19, 20]. It has been postulated that DNMT3a/b in addition to catalyzing de novo DNA methylation can facilitate demethylation by deaminating 5mC when S-Adenosyl methionine (SAM) levels are limiting [21].

The generation of TDG knockout mice has corroborated the importance of TDG in regulating active demethylation and tissue-specific gene expression. Deletion of TDG in the germline is embryonic lethal and leads to DNA hypermethylation and defects in the expression of various developmentally regulated genes [22, 23] Additionally, 5fC and 5caC levels increase five to tenfold genome wide in TDG null ES cells [24, 25]. TDG has also been implicated in transcriptional control and gene expression by functioning as a molecular scaffold protein. TDG interacts directly with ERα in a ligand dependent manner and colocalizes to the promoter of TFF1 [26] resulting in increased gene expression, effects which are lost when TDG is depleted [19]. TDG also interacts directly with other transcription factors and coregulators and in TDG null MEFs, the presence of TDG is required for recruiting the acetyltransferases CBP/p300, TET2 and other histone modifying enzymes to a subset of target genes [22, 23, 27, 28]. These findings are consistent with the notion that TDG plays a central role in epigenetic stability and methylation control.

In this study, we have generated a global profile of TDG binding in MCF7 breast cancer cells in response to E2 treatment using ChIP-Seq. We have integrated the data from our ChIP-Seq assays with data from other genomic assays to provide a global view of TDG binding. In response to E2 treatment, we show that TDG binds primarily to genomic regions upstream of target genes which, in addition to recruiting ERα and RNA Polymerase II, also bind various transcription factors, coregulators and epigenetic modifiers including p300, GATA3 and TCF7L2 and are marked by histone marks indicative of active enhancers. Importantly, TDG binds to regions which, in response to E2, transcribe eRNAs and take part in 3-dimensional restructuring of the genome. Remarkably, at a subset of enhancers that E2 targets, we found that TDG depletion abrogates E2-mediated eRNA, disrupts 3-dimensional reorganization at ER-targets such as GREB1 and disrupts E2-mediated transcription of corresponding ER-target genes. To investigate whether TDG plays a functional role in E2 signaling in breast cancer, we engineered an MCF7 TDG knockout cell line using CRISPR technology and found that TDG knockout and depletion leads to defects in E2-mediated proliferation and sensitizes MCF7 cells to the anti-estrogen, tamoxifen. Importantly, we also find that TDG depletion causes adhesion defects and drastically increases the migratory capacity and invasiveness of MCF7 cells. Collectively our findings suggest that TDG plays a central role in mediating the transcriptional and functional effects of E2 in breast cancer and may prove to be an effective therapeutic target.

## Methods

### Cell culture, treatment and transfections

MCF7 cells were obtained from ATCC and grown in low-glucose DMEM and 10% fetal bovine serum (FBS). Prior to treatment, cells were washed once with phosphate-buffered saline (PBS) and grown in phenol red-free media containing charcoal-stripped FBS (10%) for 72 h. Cells were then washed and treated with 100 nM E2 for specific time periods.

For siRNA-mediated knockdowns, cells were incubated with Lipofectamine 2000 (LifeTechnologies) and siRNA targeting TDG (Dharmacon, M-040666-01) or scrambled siRNA (Dharmacon, D-001210-03) for 24 h, media were replaced with fresh media for 48 h at which point experiments were performed.

### MCF7 CRISPR Tdg^−/−^

CRISPR Tdg^−/−^ and CRISPR Tdg^+/+^ MCF7 cells were generated as previously described using wild-type Cas-9 and two cut sites:

Cut-site 1 (bottom strand): CACCGGTTATTAAGCACTCAGTAA

Cut-site 1 (top strand): AACTTACTGAGTGCTTAATAACC;

Cut-site 2 (top strand): (CACCGTCTGGGGAATAAAAGAACAT)

Cut-side 2 (bottom strand): AAACATGTTCTTTTATTCCCCAGAC.

Primers used for detection:

Forward (GGCTGACTTGACAGGACTGA)

Reverse (CTGTGCTGAGCTGTAACGTG) [29].

### Protein extraction and western blotting

Whole-cell protein extracts were obtained by harvesting cells in RIPA lysis buffer (50 mM Tris-pH 8.0, 150 mM NaCl, 1% NP-40, 0.1% SDS and protease inhibitor cocktail), incubating on ice for 15 min, followed by centrifugation for 15 min at 4 °C (20,000 RCF). Protein concentrations were normalized, and proteins were separated using SDS-PAGE and transferred to PVDF membrane. Blocking buffer containing PBS, 0.1% Tween-20 and 5% skim milk powder was used for primary and secondary incubation as well as washes. Protein of interest was visualized using Luminata Forte Western HRP Substrate (Cat. No. WBLUF0100) and by exposure to autoradiography film (GE Healthcare).

### RNA extraction, reverse transcription and qPCR

RNA extraction was performed using TRIzol (Ambion) in accordance with the manufacturer’s directions, with additional ethanol wash steps as needed. 2 μg of total RNA was reverse transcribed using the High Capacity cDNA Reverse Transcription Kit (Applied Biosystems). mRNA levels were ascertained using pre-designed TaqMan probes (Applied Biosystems) targeting the genes of interest, while enhancer RNA levels were monitored using custom designed primers spanning the regions of interest and SYBR Green, per manufacturer’s instructions. Experiments were performed with technical triplicates and biological duplicates and run in a 96-well format using the StepOne Real-time PCR System (Applied Biosystems) using GAPDH as a normalization control, unless otherwise noted. Sequences of probes are listed in additional files (see Additional file 1).

### ChIP-Seq preparation and analysis

MCF7 cells were serum starved for 3 days, treated with 100 nM E2 for 45 min and then ChIP was performed using a polyclonal TDG-targeting antibody that was antigen affinity chromatography purified (Thermo Fisher Cat. PA5-29140), as previously described, with minor alterations [30]. Briefly, cells were cross-linked using 1% formaldehyde in PBS for 10 min under shaking at RT. 125 mM glycine in PBS was added for 5 min to quench the reaction. Cells were then washed twice with ice-cold PBS and harvested in 1 ml of ice-cold PBS buffer. The cells were then pelleted at 250 g for 10 min, washed twice with ice-cold PBS (protease inhibitors added) and then lysed using 200 μl of lysis buffer (1% SDS, 50 mM Tris–HCl [pH 8.0], 10 mM EDTA and protease inhibitors) for 15 min on ice. The cell lysate was then sonicated, and centrifuged at 15,000 rpm for 15 min. An aliquot of the supernatant mixture was saved as input DNA, and the remaining lysate was incubated with 5 μg of antibody in 50 μl of protein A/G dynabeads as per instructions. Immunoprecipitation was performed overnight at 4 °C under rotation. After the immunoprecipitation, the dynabeads were washed twice using wash buffer I (0.1% SDS, 1% Triton X-100, 2 mM EDTA, 20 mM Tris–HCl [pH 8.0], 150 mM NaCl), once with wash buffer II (0.1% SDS, 1% Triton X-100, 2 mM EDTA, 20 mM Tris–HCl [pH 8.0], 500 mM NaCl), wash buffer III (0.25 M LiCl, 1% NP-40, 1% Na-deoxycholate, 1 mM EDTA, 10 mM Tris–HCl [pH 8.0]) and twice with Tris–EDTA buffer (pH 8.0). The chromatin was eluted using 150 μl of freshly made elution buffer I (1% SDS, 0.1 M NaHCO3) twice at 65 °C for 10 min. To reverse cross links NaCl was added to the eluates and input DNA to a final concentration of 0.3 M, and both were incubated at 65 °C overnight. Immunoprecipitated DNA was purified using QIAquick PCR Purification Kit (Qiagen) and was analyzed by quantitative PCR following ChIP in technical triplicates and biological duplicates, unless otherwise noted. For high-throughput sequencing immunoprecipitated DNA was sequenced in duplicates at The Centre for Applied Genomics Next Generation Sequencing facility (Toronto, Ontario). Sequenced reads were mapped to the human genome (hg19) and Partek Genomic Suite was used to call peaks 100 bp bins at an FDR of 0.01. Peaks were further filtered, retaining only those peaks which appeared in both replicates and which showed a greater than 1.2-fold increase and had a *p* value < 0.05 in both replicates.

### MAB-Seq

Methylase-assisted bisulfite sequencing was performed according to Zhang Y et al. (2014). Briefly, 1 μg of genomic DNA was treated with 4 U of M.SssI in a 20-μl reaction containing 160 mM of SAM. After 2 h, the reaction was supplemented with an additional 4 U of M.SssI and 160 mM SAM for an additional 4 h. This was repeated three times. DNA was purified by conventional phenol/chloroform/isoamyl alcohol extraction followed by ethanol precipitation after each round of treatment. DNA was then subject to bisulfite conversion, subcloned using the TA cloning kit followed by sequencing.

### Bisulfite sequencing

DNA was extracted from MCF7 cells using Sigma’s Genomic DNA extraction kit and 1 μg was used for bisulfite conversion using the EpiTect Bisulfite Kit (QIAGEN), according to manufacturer’s instructions.

### Bioinformatics

All datasets used in this study were either based on the hg19 genome or were converted to the hg19 genome using the tool liftOver (http://genome.ucsc.edu/cgi-bin/hgLiftOver).

Mapping peaks to annotated genome was done using Cis-regulatory Element Annotation System (CEAS-Package-1.0.2, http://liulab.dfci.harvard.edu/CEAS/). To mark distance from known transcription sites, Region-gene association graphs were generated using the Genomic Regions Enrichment of Annotations Tool (version 3.0.0) and the following parameters: Association rule: Basal + extension: 5000 bp upstream, 1000 bp downstream, 1 × 106 bp max extension and curated regulatory domains included.

To determine the relative measure of similarity between E2-dependant TDG localization and that of other transcription factors we downloaded all 690 datasets from the Transcription Factor ChIP-Seq Uniform Peaks from ENCODE/Analysis at UCSC (https://genome.ucsc.edu/ENCODE/downloads.html) and determined the Fisher exact test and the pairwise Jaccard statistic using the Bedtools (2.25.0) options “fisher” and “jaccard,” with default parameters, respectively.

All motif analysis was performed using the latest version of Homer software (version 3.12) (http://homer.ucsd.edu/homer/). Peak visualization was performed using IGV (version/site) with group normalization applied where applicable.

Contrasting TDG localization with gene upregulation was done using GREAT software (using default conditions) to generate a list of genes with which TDG associates and cross-correlating this list with expression data.

GRO-Seq dataset was overlapped with sites of TDG binding or control sites (sites which contained the same sized peaks but distributed randomly using the Bedtools (2.25.0) “shuffle” option) using Homer software (v. 3.12) following software guidelines and default parameters. To generate heatmaps of looping at sites of TDG binding publicly available ChIA-PET data was overlapped with sites of TDG binding or control sites (sites which contained the same sized peaks but distributed randomly using Bedtools (2.25.0) shuffle) using deepTools2 [31].

Gene ontology analysis was completed using ConsensusPathDB (http://cpdb.molgen.mpg.de/) using default settings. Cutoff was set at *q* value > 0.05.

### Chromosome Conformation Capture (3C)

MCF7 cells were treated with 100 nM E2 for 45 min and were cross-linked using 1% formaldehyde for 10 min. The cells were then exposed to trypsin for 5 min at 37 °C followed by 5 min incubation with the 3C lysis buffer at 4 °C. 3C was then performed as previously described [32].

### Cell-to-cell adhesion

The cell–cell adhesion assay was done as previously described [33]. Briefly, plates were rinsed twice with PBS and cells were dissociated with 3 mM EDTA. Cells were collected and spun at 400 RCF for 5 min, then resuspended in DMEM media and passed through a cell strainer to dissociate cell clusters. Approximately 200,000 cells were added in the appropriate media (DMEM) onto a 6-cm petri dish. Plates were incubated at 37 °C on a shaking platform for 30 min. After this incubation period, 10 different fields of view were taken per dish at 10x objective. Clusters of > 4 cells were then counted, and counts from 10 fields of view were added together for each plate.

For cell-substrate adhesion assays MCF7 cells were grown to confluency and then treated with varying concentrations of trypsin for 2 min, at which point images were obtained. For re-adhesion assays cells were trypsinized and resuspended in full media (DMEM + 10% FBS) and re-plated onto 6 well. Images were obtained at the times documented.

### Migration and Invasion

For migration assays, transwell inserts with 8.0 um pores (Corning, Cat. No. 3422) were coated with 3 µg of gelatin and allowed to dry overnight at room temperature in a sterile environment. The following morning, the gelatin-coated filters were reconstituted with 100uL serum-free DMEM for 90 min on a shaker. For invasion assays, transwell inserts with 8.0 um pores were coated with 100 µL of 1 mg/mL Matrigel (Corning, Cat. No. 356234) and incubated at 37 °C for one hour to allow for solidification of the Matrigel layer. For both assays, MCF7 cells transiently transfected with siC or siTDG RNA for 3 days prior to being treated with or without tamoxifen. 3 days after treatment, cells were harvested using 3 mM EDTA. A cell suspension of 100,000 cells in DMEM + 0.1% BSA was added to the upper well of each transwell insert, and 750uL of DMEM + 10% FBS was added to the lower chamber as a chemoattractant. The cells were incubated at 37 °C and allowed to migrate or invade for 72 h. After this incubation period, the transwell membranes were fixed with 1% glutaraldehyde for 20 min, followed by a 15-min stain with full strength hematoxylin, and brief dip in 1% ammonium hydroxide. Non-migrating and non-invading cells were wiped off the upper surface of the membrane with a cotton swab. Images of 3 non-overlapping fields of view per well were acquired using Image-Pro Analysis Software on an inverted microscope at 10× objective. Cells were counted using ImageJ. Means derived from four replicates were used during analysis.

## Accession numbers

All accession numbers are present in additional files (see Additional file 2).

## Results

### Global TDG binding in response to E2

**Fig. 1:**
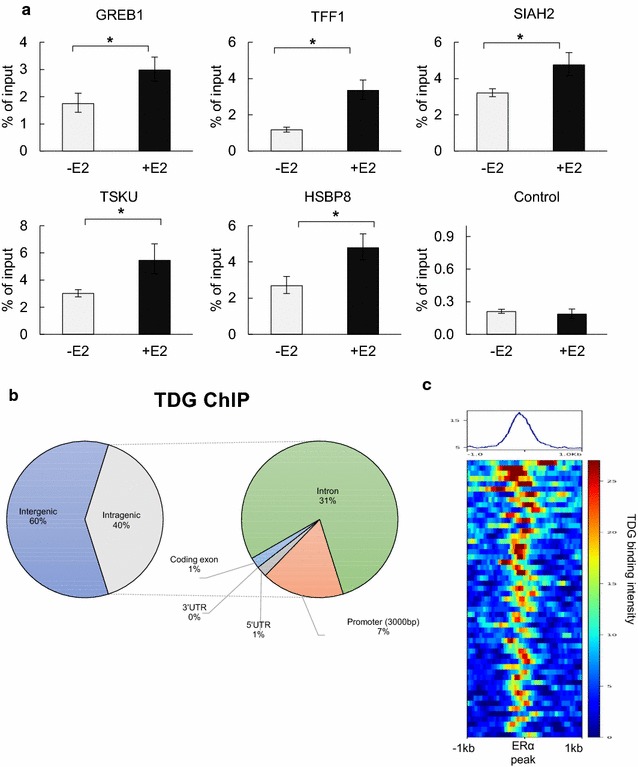
Global analysis of E2-dependent TDG localization. **a** MCF7 cells were treated with 100 nM of E2 (45 min) and ChIP-qPCR was performed using TDG antibody. Region used as negative control shows low level of TDG binding in ChIP-Seq data with no change in levels after E2 treatment. (*p value < 0.05, error bars represent standard deviation of the mean, *n* > 2). **b** Sites of E2-dependent TDG binding were mapped to the annotated genome using CEAS. **c** Sites of ER binding (∓ 1000 bp) overlaid with TDG binding signal, showing strong relationship between location of TDG binding and ER binding at these regions

Previous reports have shown that, in response to E2, TDG physically interacts with ERα and localizes to TFF1/PS2, a well-characterized ER-target gene [19]. To determine whether colocalization of TDG and ERα extends to other genomic locations, MCF7 cells were treated with 100 nM E2 for 45 min and ChIP-Seq was performed using a TDG-specific antibody. Biological replicates were performed, and for each replicate the reads were processed to remove duplicates and corrupted reads before being mapped to the human genome (hg19). Areas of significant enrichment were identified using an FDR of 0.01. To identify only highly confident peaks, the peak set was filtered and only those peaks with a *p* value < 0.05 and fold change greater than 1.2-fold was retained. Finally, by retaining only peaks which appeared in both biological replicates we were able to identify 117 highly confident regions to which TDG localized in response to E2 (Additional File 3A and B). Validations were performed using conventional ChIP-qPCR (Fig. 1a). Compared to genomic background, global analysis of the high-confidence TDG peaks revealed that E2-dependent TDG binding was enriched at promoters (7% of total TDG peaks, compared with 3% genomic background) as well as distal to promoters with approximately 60% occurring intergenically (compared to 52% for background) (Fig. 1b and Additional file 3C). However, overlapping TDG peaks with sites of E2-dependent ERα localization revealed that 45% of TDG peaks occur at the same sites where ERα localizes in response to E2 (Fig. 1c and Additional file 3D)

**Fig. 2:**
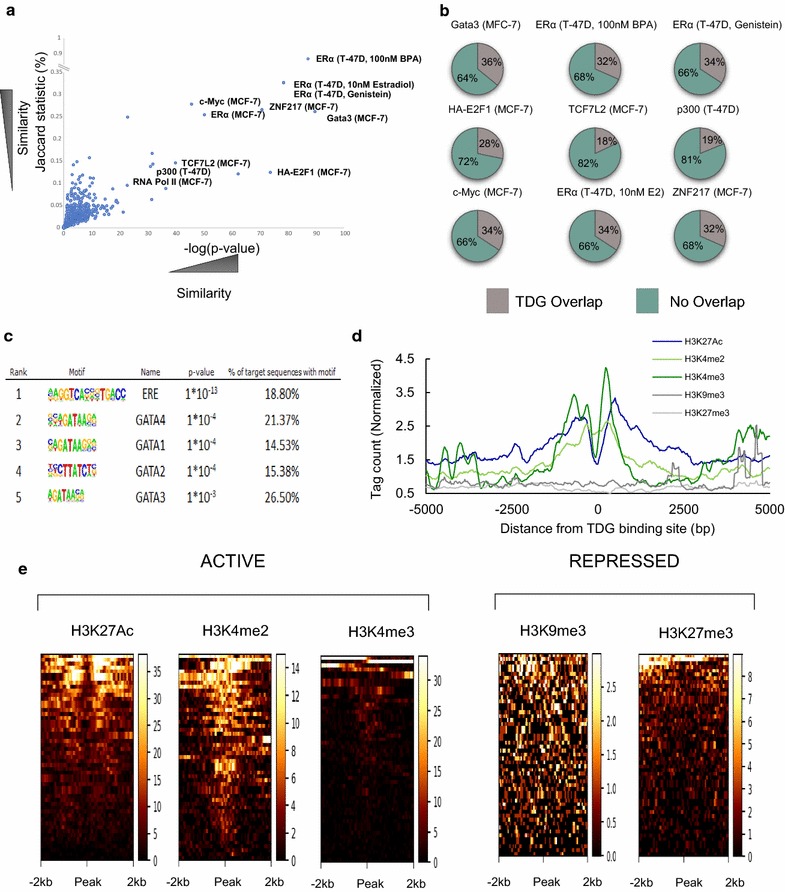
TDG localizes to sites occupied by transcription factors. **a** Individual datasets obtained from the Transcription Factor ChIP-Seq Uniform Peaks dataset from ENCODE were compared to the TDG dataset using the Fisher’s exact test p value and Jaccard statistic to determine relative similarity. A subset of the most similar matches is labeled with the cell type and treatment, if disclosed, in brackets. **b** Overlap between TDG peaks and the corresponding ENCODE dataset (The average binding profile of the 10 least similar datasets from the ENCODE database was used as controls. None of the 10 least similar had more then a single peak which overlapped with our dataset). **c** Motif analysis performed on sites of E2-mediated TDG localization revealed the canonical ERE as a top hit followed by GATA protein consensus binding site. **d** Overlap of ChIP-Seq signal from publically available histone datasets at sites of E2-dependent TDG binding in MCF7 cells. **e** Heatmaps showing intensity of histone marks at sites of TDG binding. Sites where TDG localizes in response to E2 are enriched for histone marks indicating “active” enhancers (H3K27Ac, H3K4me2 and H3K4me3) while depleted for those marking “repressed” enhancers (H3K9me3 and H3K27me3)

Recent studies have shown that the ERα is found at enhancers and colocalizes with various transcription factors known to play important roles in enhancer regulation [34]. The publicly available database “Transcription Factor ChIP-Seq Uniform Peaks” from ENCODE contains the binding profiles of these and other transcription factors from numerous cell lines. We compared E2-dependent TDG binding to the 690 files available from ENCODE using two measures of similarity: Jaccard statistic and the Fisher exact test. Within the ENCODE datasets, those which most closely resemble E2-dependent TDG binding are the datasets from experiments recording ERα binding in response to E2 treatments in breast cancer cells such as MCF7 cells and the metastatic T-47D breast cancer cell line. The other transcription factors that exhibit binding patterns most similar to that of TDG are p300, GATA3, TCF7L2, the oncoprotein ZNF217 and RNA polymerase II (Fig. 2a, b and Additional file 4). Importantly, these proteins have been identified as having important roles at enhancers. Interestingly, we also observe higher similitude between TDG and both Myc and E2F1. While both proteins have been implicated in breast cancer progression, little is known concerning their respective roles at specific enhancers. Motif analysis focusing on TDG peaks that overlap with ER revealed an enrichment for the canonical ERE motif, as well as the GATA DNA binding motif (Fig. 2c and Additional file 5). In contrast, TDG peaks that do not overlap with ER are enriched for only a single motif, PU.1 (Additional file 6). Transcription factors from the GATA family regulate genes that are implicated in cell cycle arrest and cell survival [35]. GATA3, specifically, has been identified as a critical component of mammary epithelial cells development and is 1 of 3 genes that have been shown to be mutated in > 10% of breast cancers [36]. Furthermore, GATA3 has been shown to mediate enhancer accessibility in MCF7 cells and its depletion results in an altered binding profile of ERα upon E2 treatment, with a corresponding altered change in target gene expression [36].

Distal regulatory sites involved in gene repression or activation are frequently marked by specific histone modifications. Enhancers involved in transcriptional activation have high levels of H3K27Ac, H3K4me2 and H3K4me3 [37–39], while sites involved in silencing are often devoid of most of these marks and instead contain H3K9me3 and H3K27me3 [40, 41]. To gain a better understanding of the epigenetic makeup of sites to which TDG localizes in response to E2, we compared our data with that of publicly available ChIP-Seq datasets performed using antibodies against histone modifications. Aggregate plots and heatmaps at sites of TDG, or TDG and ER, localization in response to E2, revealed that TDG localizes with histones containing marks found at active enhancers (H3K27Ac, H3K4me2 and H3K4me3) and depleted almost entirely of histone markings corresponding to repressed or silenced enhancers (H3K9me3 and H3K27me3) (Fig. 2d, e).

**Fig. 3:**
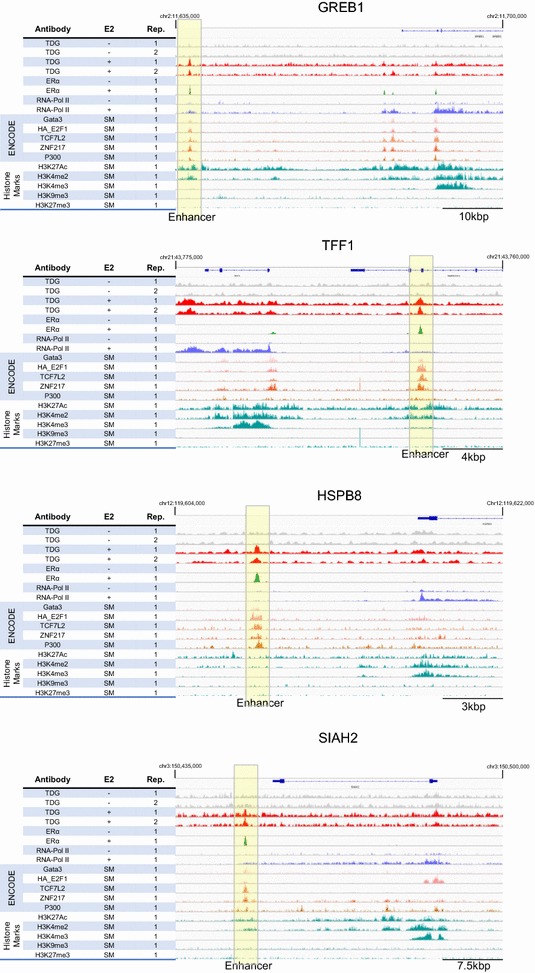
Transcription factor binding and histone modifications at a subset of TDG-targeted genes. Genomic regions surrounding genes identified as having E2-dependent TDG binding and increased transcription show precise overlap of TDG, ER and a subset of ENCODE transcription factors. Additionally, overlap with datasets containing histone ChIP-Seq data reveals the enrichment of histone marks corresponding to active/poised enhancers and depleted of those markings found at repressed/silenced enhancers (*SM* standard media)

To determine the extent of overlap of TDG and the transcription factors identified in our global analysis, we cross-referenced genes whose transcription is induced upon E2 treatment with genes that are adjacent to TDG peaks (∓ 100 kb) to identify E2-inducible genes that are potentially regulated by TDG (Additional file 7). A subset of genes meeting these criteria was selected, and a closer examination of the genomic landscape surrounding sites of TDG was performed (Fig. 3 and Additional file 8). Remarkably, we find highly enriched binding occurs precisely at sites which bind the transcriptional factors identified in our original ENCODE analysis. Furthermore, these sites of E2-dependent TDG localization are enriched for histone marks that correspond to active enhancers while being devoid of marks corresponding to repressed/silenced enhancers, predicted by our previous bioinformatic analysis (Fig. 2d, e). We also found a basal-level of TDG binding across the DNA at these regions which is likely reflective of TDG’s non-specific DNA binding activity.

### TDG depletion disrupts E2-mediated transcription of ERα target genes

**Fig. 4:**
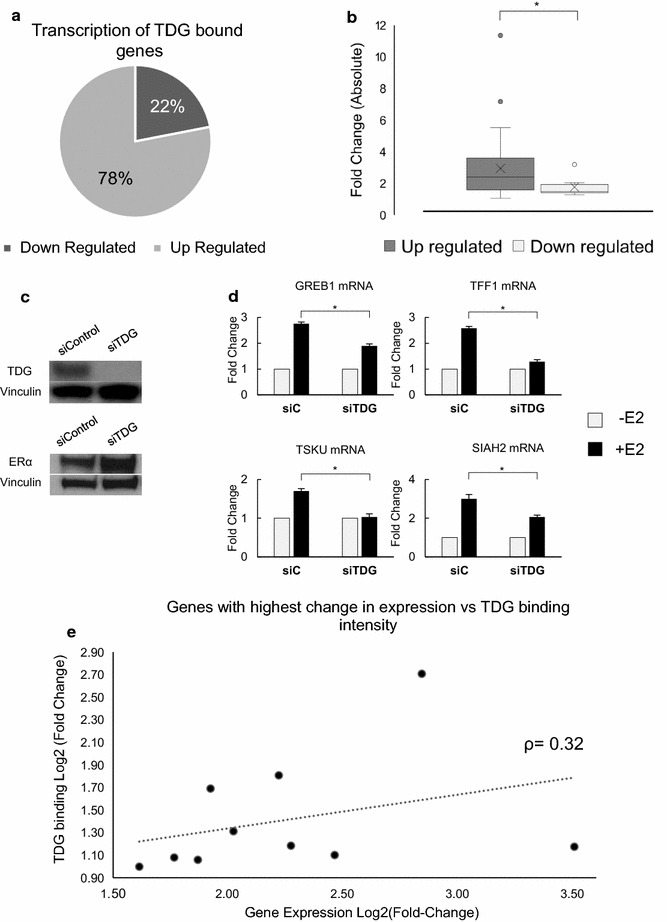
TDG is required for E2-dependent gene expression. TDG peaks were mapped to genes using GREAT software and cross-referenced with publicly available MCF7 E2-dependant expression data. **a** Most genes associated with TDG binding undergo upregulation in response to E2 and **b** the fold change experienced by upregulated genes in response to E2 is greater in magnitude than the fold change experienced by downregulated genes (box-and-whisker plot). **c** Western blot of TDG and ER levels after treatment with scrambled siRNA (siControl) or siRNA targeting TDG (siTDG) (left panels). **d** To determine whether TDG was important for transcriptional upregulation of these genes, siControl or siRNA targeting TDG was treated with 100 nM E2 for 1 h. Analysis of mRNA levels using qPCR revealed that loss of TDG decreases, and in some cases completely abrogate, E2-mediated transcription (*n* = 3, *p* value < 0.05). **e** Comparison of E2-mediated TDG binding at genes which also experience the greatest increase in E2-mediated transcription (Spearman coefficient shown)

Due to its role as a transcriptional coactivator, we sought to determine whether genes to which TDG binds, in response to E2, are up- or downregulated. To address this, we identified genes adjacent to TDG binding sites and then obtained their transcriptional response to E2 from publicly available data (Additional file 7) [42]. We find that genes which are differentially expressed in response to E2, and to which TDG localizes, are most often upregulated and the magnitude of change is significantly higher in genes that are upregulated when compared to those that are downregulated (Fig. 4a, b and Additional file 7). To determine whether TDG is critical to the E2-dependant changes in expression, we first treated MCF7 cells with siRNA targeting TDG and immunoblotted for TDG and ER to ensure ER levels remained stable during TDG depletion. We then treated MCF7 cells previously treated with scrambled siRNA or siRNA targeting TDG with 100 nM E2 for 1 h and measured mRNA levels of a subset of target genes using qPCR. Remarkably, we find that TDG depletion does not affect levels of ER, yet significantly reduces E2-mediated increase in the transcript levels of all ER-dependent target genes tested (Fig. 4c, d). A look at the top 10 genes that bind both TDG and are expressed upon E2 treatment reveals a slight correlation between magnitude of TDG binding and gene expression (Fig. 4e and Additional file 9).

### TDG is required for eRNA production

**Fig. 5:**
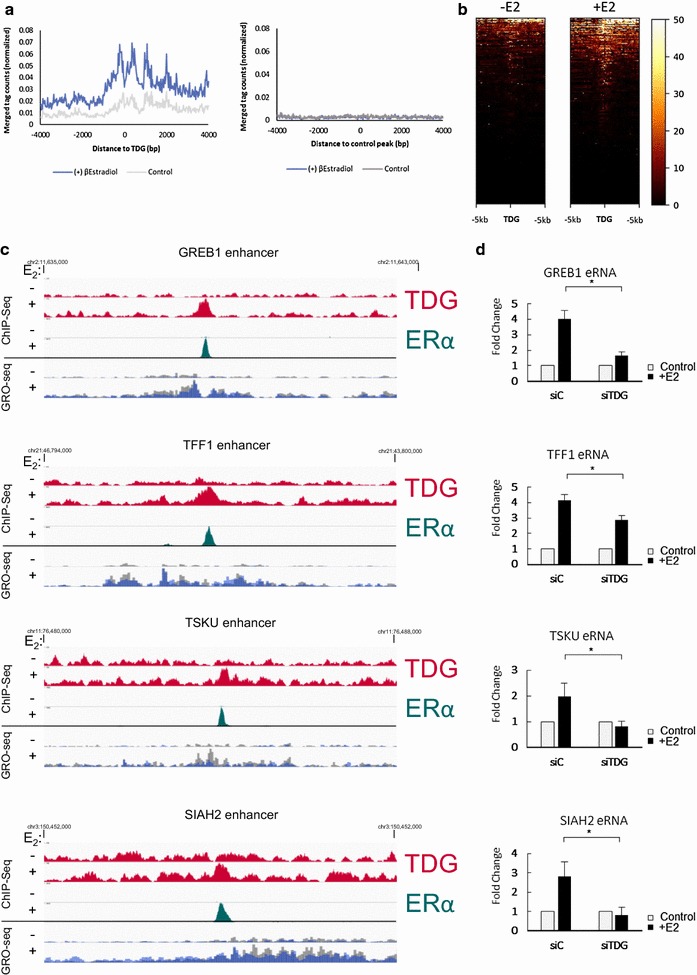
TDG depletion impacts eRNA production. **a** Publically available GRO-Seq data looking at levels of transcription at sites of E2-dependent TDG binding reveal that sites of TDG binding experience an increase in transcription in response to E2 (a set of random peaks reflecting precisely the size distribution of actual TDG peaks was used as control). **b** Heatmap of nascent transcription using publically available GRO-Seq data at sites of TDG ∓ E2. **c** E2 effects on localization of TDG and ER, as well as transcription response, at specific targets. **d** To determine whether TDG is required for eRNA production, MCF7 cells were depleted of TDG using siRNA and transcript levels were measured in response to 100 nM of E2 (1 h) (qPCR, *p* value < 0.05)

Recent studies using global run-on sequencing (GRO-Seq) characterized nascent transcription in response to E2 treatment in MCF7 cells and showed that many of the ERα bound enhancers bind RNA pol II and transcribe enhancer RNAs (eRNA) [4]. Importantly, eRNA transcription and/or eRNA transcripts per se are required for activation of adjacent target genes [4]. To determine whether TDG plays a role in eRNA transcription, we first looked too see whether sites of TDG binding coincide with sites of E2-mediated eRNA transcription in MCF7 cells by overlaying sites of E2-dependent TDG localization with publicly available GRO-Seq data. We find that, on average, sites of E2-dependent TDG localization also undergo a concomitant increase in transcription in response to E2 (Fig. 5a, b). Furthermore, sites of TDG binding at the enhancers of target genes we examined previously overlap precisely with locations that undergo transcription at those targets (Fig. 5c). Transcription of noncoding RNA from ER-targeted enhancers is readily induced by 100 nM E2 treatment for 1 h. Remarkably, depleting TDG protein using siRNA prior to treatment abrogates the ability of E2 to induce eRNA from TDG-targeted enhancers (Fig. 5d and Additional file 10). These findings reveal for the first time a potential mechanism by which TDG regulates ER-signaling.

### TDG is required for 3D conformational changes

**Fig. 6:**
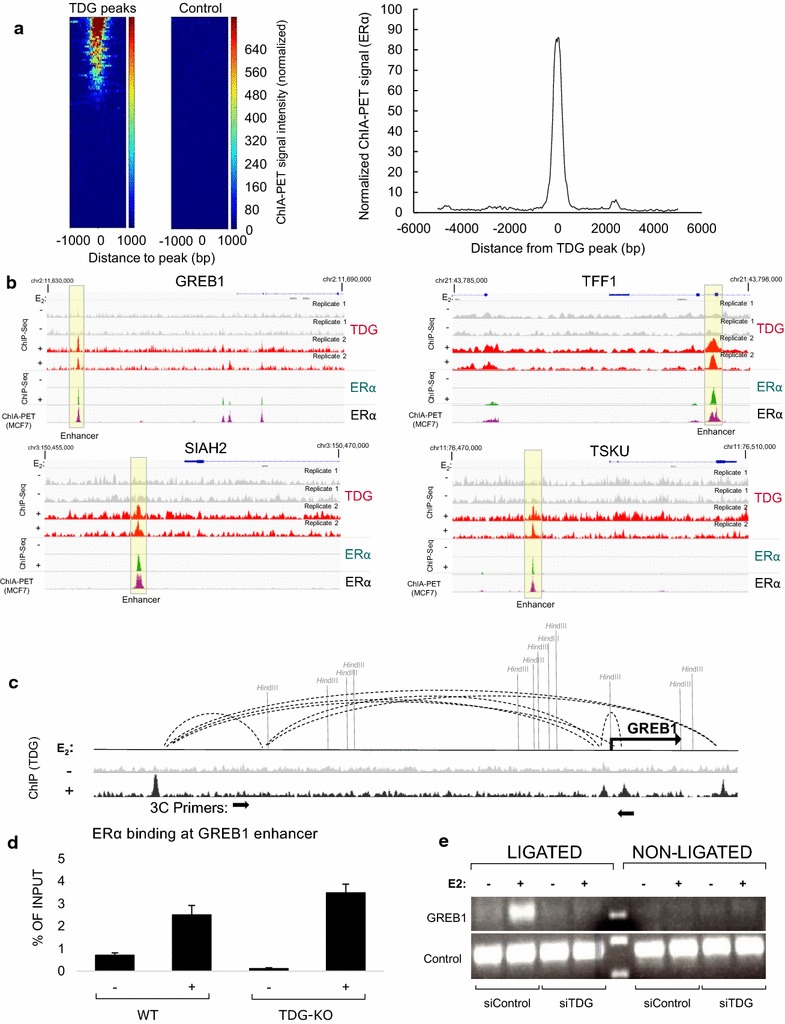
TDG peaks overlap with sites involved in promoter-enhancer looping. TDG binding was compared to public datasets containing E2-dependent ERα localization and ChIA-PET performed using an antibody against ERα. **a** Heatmap showing ChIA-PET signal at sites of TDG binding (left) and aggregate plot showing global average (right). **b** Overlap of TDG, ER and ChIA-PET signal at specific sites reveals TDG, ER and looping occur at precisely the same locations at these sites. **c** Schematic of GREB1 showing approximate locations of looping as identified by publically available data. **d** ChIP using ER in the presence and absence of TDG as well as − or + E2, showing that ER binding is unaltered during depletion of TDG. **e** Loss of TDG prevents enhancer–promoter looping at the GREB1 locus. MCF7 cells were treated with siControl or siTDG, and then treated with 100 nM E2 for 1 h. 3C, semiquantitative method of measuring the looping between the GREB1 enhancer and promoter, revealed that E2-driven looping of the enhancer and promoter is disrupted upon TDG knockdown

Recent work in both ER and androgen receptor-mediated signaling has revealed that eRNA transcription and/or eRNA transcripts facilitate 3-dimensional reorganization of the genome bringing the enhancer regulatory region into proximity with the promoter and activating optimal target gene transcription [4, 5]. Chromatin Interaction Analysis by Paired-End Tag sequencing (ChIA-PET) is a technique used to capture and quantitate long-range chromatin interactions that occur in the presence of a protein of interest. By comparing ER-dependent TDG binding to data obtained from ChIA-PET looping that occurs at sites of ERα binding in MCF7 cells, we find that a large component of E2-mediated TDG binding occurs precisely at genomic sites that are involved in the interactions between promoter and enhancer (Fig. 6a, b). Previous groups have reported that eRNA production at GREB1 is a critical mediator of long-range looping and targeted eRNA degradation is itself enough to attenuate looping and enhancer complex formation at this gene (Fig. 6c) [4]. Based on our findings that TDG depletion inhibits E2-driven production of eRNA at GREB1, we predicted that TDG depletion may negatively impact the long-range loop formation. To explore this possibility, we induced formation of looping at GREB1 by treating cells with 100 nM E2 for 1 h after siRNA-mediated depletion of TDG. Similar to previous reports we find that E2 is able to induce the formation of the enhancer–promoter loop at GREB1. Remarkably, ER continues to be recruited to enhancer in the absence of TDG (Fig. 6d), but 3D reorganization is abrogated when TDG is depleted, highlighting TDG’s impact not only on eRNA production but also on 3-dimensional chromosomal rearrangement (Fig. 6e).

### Methylation status is not impacted by TDG knockdown

**Fig. 7:**
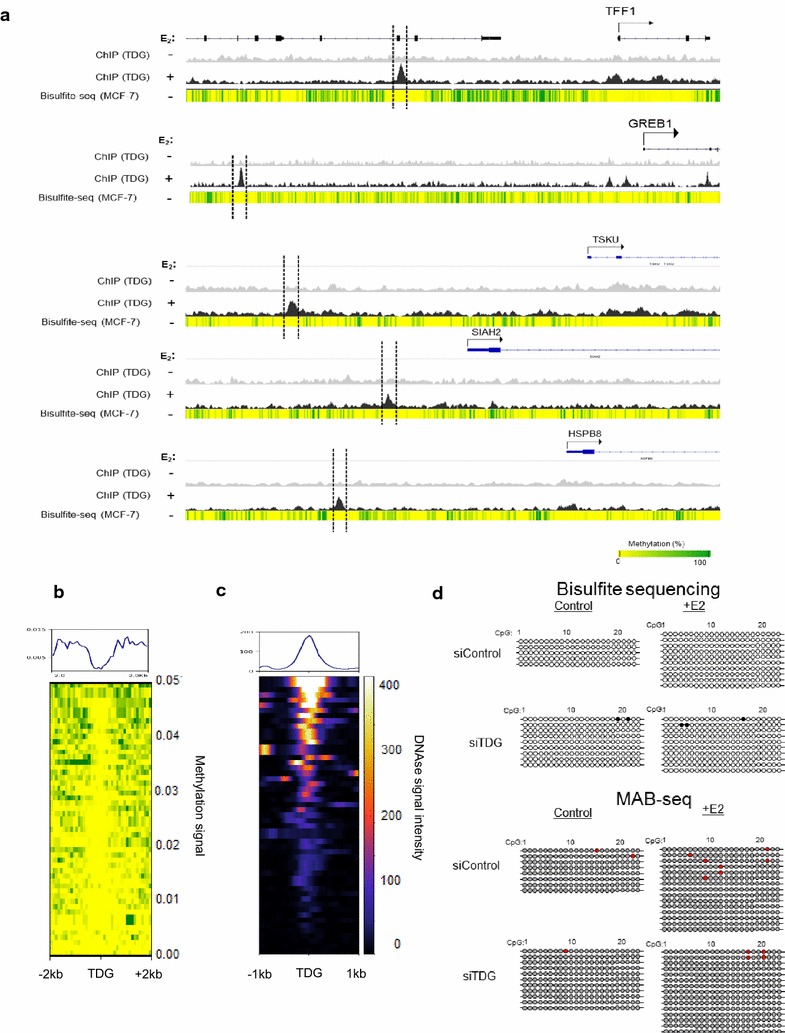
Cytosines in TFF1 and GREB1 enhancers are epigenetically unmodified. **a** Comparing TDG binding at sites regulated by E2 reveals that TDG binds to hypomethylated locations. **b** Heatmap comparing methylation signal at sites of TDG binding globally reveals that TDG binds to hypomethylated sites. **c** MCF7 DNase signal intensity, revealing that, globally, TDG binding occurs preferentially at sites which are hypomethylated and “open.” **d** Bisulfite sequencing and MAB-Seq at TFF1 after cells were treated with scrambled siRNA (siControl) or siRNA targeting TDG (siTDG) and then with or without E2 treatment. TFF1 enhancer is devoid of any methylation or active demethylation metabolites and remains so in response to E2 or TDG depletion

Recent studies have shown that the 5mC derivatives 5caC and 5fC, generated during active demethylation, accumulate at “open” enhancers in TDG knockout MEFs [25, 43] suggesting that active demethylation may be important for eRNA production. Comparing sites of E2-dependent TDG binding with publicly available bisulfite sequencing and DNase data revealed that while TDG binding coincides with “open” genomic regions, the CpGs are not methylated and the regions are in an “open” state (Fig. 7a–c) [44]. Bisulfite sequencing is unable to distinguish between unmethylated cytosine and 5fC/5caC. To obtain a clearer picture of what impact E2 signaling and TDG may have on 5mC derivatives at enhancers, we performed methylase-assisted bisulfite sequencing (MAB-Seq). MAB-Seq consists of pre-treating genomic DNA with the bacterial methyltransferase enzyme *M.SssI*, which methylates unmodified cytosines (C). The *M.SssI*-treated DNA is then treated with bisulfite which converts 5fC and 5caC to thymine (T) but does not convert Cs (which have been converted upstream to 5mC by *M.SssI*). Therefore, sequencing would indicate 5fC and 5caC as T, whereas C/5mC/5hmC would be sequenced as C. Comparing bisulfite sequencing with MAB-Seq results confirms that TFF1 and GREB1 enhancers are composed almost entirely of unmethylated cytosines and are not altered either in response to E2 treatment or TDG depletion, indicating that active demethylation at specific enhancers is not required for E2 signaling (Fig. 7d and Additional file 11).

### TDG knockdown affects cell proliferation of MCF7 breast cancer cells

**Fig. 8:**
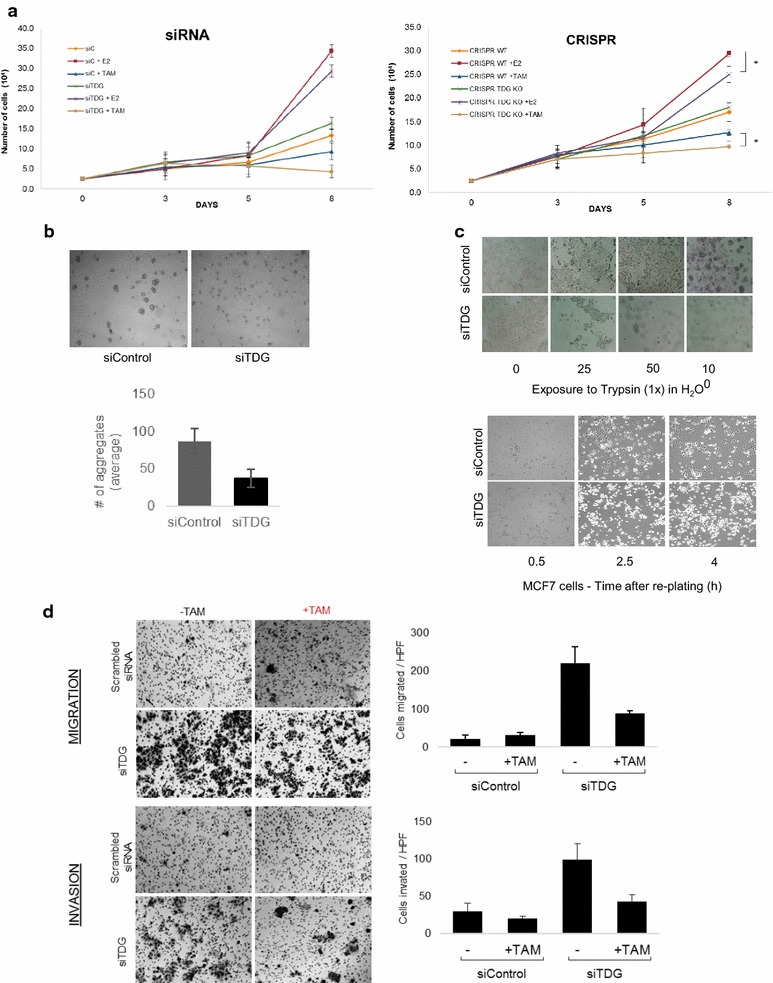
TDG depletion sensitizes MCF7 cells to Tamoxifen and produces proliferation and attachment deficiencies. **a** Growth curves examining responsiveness of CRISPR-mediated (right panel) and siRNA-mediated (left panel) TDG knockout and knockdown, respectively. MCF7 cells were grown for 48–72 h in charcoal-stripped phenol red-free media prior to treatment (left panel) (*n* = 3, *p* value < 0.05, error bars show ∓ 1 standard deviation). **b** To examine migration and adhesion, MCF7 cells were treated with either scrambled siRNA or siRNA targeting TDG and grown in regular media for 2 days prior to performing the cell-to-cell adhesion assay. We found that MCF7 s with depleted levels of TDG form significantly less aggregates with one another (*n* = 4, error bars indicate standard deviation ∓ 1). **c** Treatment of MCF7 cells with varying concentration of trypsin revealed that those depleted of TDG detached from tissue-plate substratum at lower concentrations (top panel) and upon resuspension TDG-depleted cells demonstrated re-attachment deficiencies, remaining loosely attached and spherical while cells treated with siControl became stabilized with visible “flattening” indicative of cell-substratum contacts(bottom panel). **d** To measure the effect of TDG on the migration and invasion capacity, TDG was depleted in MCF7 cells using siRNA which were treated with or without Tamoxifen for 3d. Cells were harvested using 3 mM EDTA, and a suspension of 100,000 cells was incubated at 37 °C for 72 in gelatin or Matrigel to test migration and invasion, respectively. Cells were counted using ImageJ (*n* = 4). MCF7s depleted of TDG show a drastically more aggressive profile with cells migrating and invading gel at a significantly higher rate than siControl cells. Tamoxifen treatment had an impact on the increased aggressiveness, although failed to restore cells to basal capacity

Gene ontology (GO) enrichment analysis was performed on genes which bind TDG and are upregulated in response to E2. We found a significant enrichment at GO terms directly related to proliferation, including “regulation of epithelial cell proliferation,” as well as multiple terms implicating a role in “differentiation” and “Wnt signaling” (Additional file 12). Wnt signaling was a particularly interesting finding as TDG has recently been shown to directly upregulate components of Wnt signaling pathway in colorectal cancer (CRC), and TDG depletion inhibited proliferation of CRC cells both in vitro and in vivo [45]. To determine whether TDG plays a role in E2-dependent cell proliferation we deleted TDG from MCF7 cells constitutively using CRISPR technology, or transiently using siRNA (Additional file 13). In both systems, TDG depletion significantly decreases the E2-mediated increase in proliferation compared to control cells, while also increasing sensitivity to the anti-estrogen tamoxifen, with TDG-depleted cells exhibiting a stronger cytostatic response than controls (Fig. 8a).

Estrogens and the anti-estrogens, such as tamoxifen, can modulate the proliferation capacity of breast cancer cells in part by causing complex rearrangements of both the cytoskeleton and adhesion apparatus [46–48]. Remarkably, we find that TDG depletion in MCF7 cells drastically decreases their ability to adhere to the substratum, and to one another (Fig. 8b, c). This finding is important as anti-estrogens promote an invasive phenotype in breast cancer cells which have adhesion deficiencies [49]. To test whether the adhesion defects observed in TDG-depleted MCF7s promote migration or invasion, we depleted TDG in MCF7 s using siRNA and treated cells with the anti-estrogen tamoxifen and recorded their ability to transverse gelatin, or Matrigel, respectively. While TDG depletion sensitizes MCF7 cells to the cytostatic effects of tamoxifen, we find that MCF7 cells depleted of TDG become much more aggressive, with significant increases in both migration and invasion capacity (Fig. 8d).

## Discussion

Utilizing a combination of functional genomic analysis and biological assays, we have identified a role for TDG in E2-dependant signaling in MCF7 breast cancer cells. In response to E2 TDG localizes to distal regulatory sites of ER-target genes. Approximately half of the TDG binding sites identified overlap with sites of E2-mediated ER binding. Importantly, in response to E2 TDG localizes to enhancer regions that play an important role in the production of eRNAs and 3-dimensional reorganization important for target gene transcription. By focusing on a subset of TDG target genes whose transcription is upregulated in response to E2 treatment, we found that TDG depletion significantly reduces the ability of E2 to induce transcription of eRNA produced at the enhancers, disrupts looping and inhibits transcription of the target genes.

eRNA-producing enhancers have several common characteristics that include increased binding of transcriptional coactivators, greater chromatin accessibility and increased formation of enhancer–promoter looping. Although a direct functional role of eRNAs is still unclear, mounting evidence supports the notion that eRNA production is not merely transcriptional noise as previously suggested but plays a functional role by contributing to transcriptional activation of adjacent coding genes. While it remains unclear as to whether eRNA transcription or the eRNA transcripts per se are responsible for the 3-dimensional reorganization that brings the enhancer and promoter into proximity of one another, we have found that TDG depletion disrupts gene transcription broadly, interrupting both eRNA production, 3-dimensional reorganization and activation of target gene transcription. Furthermore, the finding that TDG binding occurs primarily outside of promoters suggests that dynamics at enhancers play an important role in regulating ER-target gene expression.

Previous reports have suggested that stimulation of ER-signaling at some promoters triggers a cyclical methylation/demethylation mechanism involving DNMTs [19, 20]. It was proposed that in addition to functioning as DNA methyltransferases, DNMT3a/b is capable of deaminating 5mC when SAM is limiting. The resulting G/T mispair is then excised by TDG and the base excision repair machinery restores unmethylated cytosine. More recently, reports have emerged showing that TDG depletion in embryonic stem cells resulted in the accumulation of active demethylation metabolites 5fC and 5caC at identified enhancer regions [24, 50]. To determine whether active demethylation plays a role at enhancers we used MAB-Seq to establish a profile of the active demethylation intermediates 5fC/5caC at the site of TDG binding pre- and post-E2 treatment and in conjunction with wild-type TDG levels or with siRNA-mediated depletion of TDG. We found that the TFF1 enhancer appears to be composed entirely of unmodified cytosines regardless of E2 treatment. The observation that TDG depletion in MCF7 breast cancer cells leads to no accumulation of 5fC/5caC supports reports that the glycosylase activity of TDG is dispensable for E2-mediated signaling and instead it is TDGs ability to act as a coactivator that potentiates ER activity [26].

The importance of E2-dependent signaling in breast cancer has been well documented. For example, it has been demonstrated that growth, proliferation and metastatic nature of MCF7 cells transplanted into nude mice are E2 dependent [51]. Treatment of MCF7 s with either E2, or the anti-estrogen tamoxifen, has been shown to cause changes in proliferation and growth through complex large-scale rearrangements of the cytoskeleton and adhesion apparatus [47, 49, 52]. Based on our findings that TDG seems to be intimately involved with E2 signaling we investigated whether its role extends to proliferation. We found that deleting TDG from MCF7 cells using either CRISPR technology or siRNA transfection inhibited E2-dependent proliferation. Interestingly, GO analysis revealed that E2 causes TDG binding and upregulation of genes involved in “Wnt signaling,” in addition to other proliferation-related categories such as multiple GO terms referencing “differentiation.” This is consistent with previous studies showing that TDG plays a critical role in the progression of colorectal cancer by upregulating components of Wnt signaling pathway in a CBP/p300-dependent manner. Importantly, researchers observed that TDG depletion significantly inhibited proliferation of CRC cells in vitro and in vivo [45]. Taken together, our findings suggest that TDG’s role in Wnt signaling may perhaps extend outside of CRC and play an important role in breast cancer. Further studies will be required to determine to what extent this may be the case.

We have found that TDG is also critical for maintaining proper cell–cell and cell-substratum contacts in MCF7 cells and depletion of TDG leads to broad adhesion defects. This is an important consideration as tamoxifen treatment has been shown to promote an invasive phenotype in ER-positive breast cancers when cell–cell contacts are weak [49]. Migration and invasion assays have confirmed this, revealing that TDG depletion results in a much more aggressive phenotype with cells demonstrating drastically increased migration and invasion capacity in response to tamoxifen suggesting that TDG possesses tumor suppressive properties despite being a positive regulator of estrogen-dependent cell growth. Based on these opposing roles we would predict that cells containing a TDG mutation would not have a selective growth advantage and would be removed before causing genetic and/or epigentic changes resulting in cancer. This may explain why homozygous mutations for TDG have not been identified in breast cancer based on TCGA dataset analysis. Taken together, our findings reveal TDG is important in E2 signaling by regulating eRNA production at ER-targeted enhancers. Furthermore, our functional analysis revealed that TDG plays a critical role in proliferation in response to estrogens and anti-estrogens. Further investigation into the potential for TDG as a therapeutic target is strongly warranted.

## Additional files

**Additional file 1.** Primers used in study.

**Additional file 2.** External file sources used in study.

**Additional file 3.** ChIP-Seq treatment and processing. (A) Reads were mapped to the human genome (hg19) and peaks were built using Partek Genomic Suite. Peaks which occurred in both replicates were filtered based on p value (0.05) and fold change (1.2 fold) to build the final set of 117 high-stringency peaks. (B) Distribution of fold changes (Treatment/Control), peak lengths and p values. (C) Genomic features of hg19 genome build (D) Venn diagram showing overlap between E2-dependent TDG peaks and ER peaks obtained from public dataset.

**Additional file 4.** Comparison of TDG peaks ENCODE Transcription Factors.

**Additional file 5.** Motif analysis containing all sites of TDG binding.

**Additional file 6.** Motif analysis performed on sites of E2 mediated TDG localization that do not overlap with ER binding, revealed enrichment only for single motif.

**Additional file 7.** Gene-list of targets which bind TDG and are regulated by E2.

**Additional file 8.** Genomic landscape at TSKU. Genomic region at E2-dependent TDG binding site of TSKU, a E2-mediated target gene. Shown are TDG, E2, ENCODE subset of transcription factors and histone marks.

**Additional file 9.** E2 dependent transcription of genes bound by TDG (A) Transcription levels in response to E2 at genes which bind both TDG and ER. (B) Relationship between TDG binding intensity and gene expression reveals a weak relationship between how much TDG is recruited and gene expression.

**Additional file 10.** eRNA production at GREB1 and TFF1. MCF7 cells were depleted of TDG using siRNA, and transcript levels were measured in response to 100 nM of E2 (1 h) (qPCR, *p* value < 0.05).

**Additional file 11.** MAB-Seq of GREB1 enhancer region. Cytosines in the GREB1 enhancer region which are mostly unmodified, regardless of TDG status or E2 treatment.

**Additional file 12.** GO Analysis on gene-list of genes which are regulated by E2 and targeted by TDG in response to E2.

**Additional file 13.** CRISPR-mediated deletion of TDG in MCF7 cells. (A) To eliminate TDG protein from MCF7 cells we used the CRISPR/Cas-9 and pair guided excision to remove a 490 bp region of TDG which contained exon 2 and which also created a frame-shift. (B) Protein levels of TDG in wild-type (WT) cells as well as those edited using CRISPR or siRNA (C: control siRNA or nontargeting CRISPR, TDG: siRNA targeting TDG or CRISPR targeting TDG).

## Abbreviations

5caC: 5-Carboxylcytosine
5fC: 5-Formylcytosine
5hmC: 5-Hydroxymethylcytosine
5mC: 5-Methylcytosine
AID: Activation-induced deaminase
APOBEC 1-4: Apolipoprotein B mRNA editing enzymes
BER: Base excision repair pathway
ChIA-PET: Chromatin Interaction Analysis by Paired-End Tag sequencing
CpG: Cytosine–guanine dinucleotide
CRC: Colorectal cancer
DNMT: DNA methyltransferases
E2: β-estradiol
eRNA: Enhancer RNAs
ERα: Estrogen receptor α
ERβ: Estrogen receptor β
FBS: Fetal bovine serum
GO: Gene ontology
GRO-Seq: Global run-on sequencing
MAB-Seq: Methylase-assisted bisulfite sequencing
PBS: Phosphate-buffered saline
TDG: Thymine DNA glycosylase
TET: Ten eleven translocation
