# Supplementary figures and images for: Genome-wide analysis reveals a role for TDG in estrogen receptor-mediated enhancer RNA transcription and 3-dimensional reorganization
Source: Epigenetics Chromatin. 2018 Jan 29;11:5. doi: 10.1186/s13072-018-0176-2 (PMC5787930; doi:10.1186/s13072-018-0176-2)

A

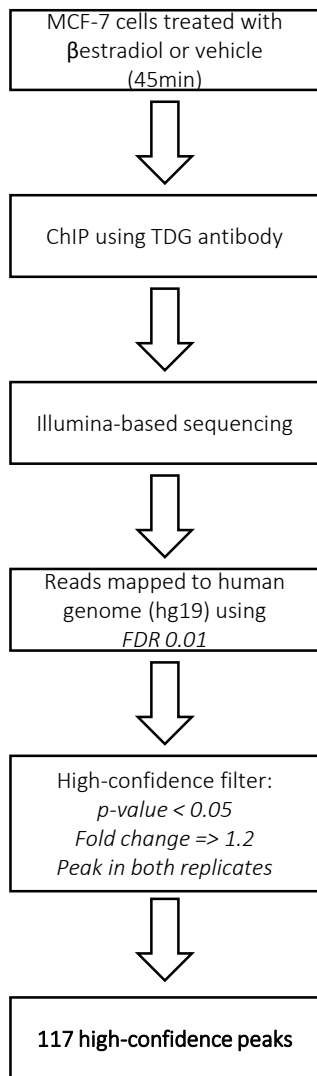

B

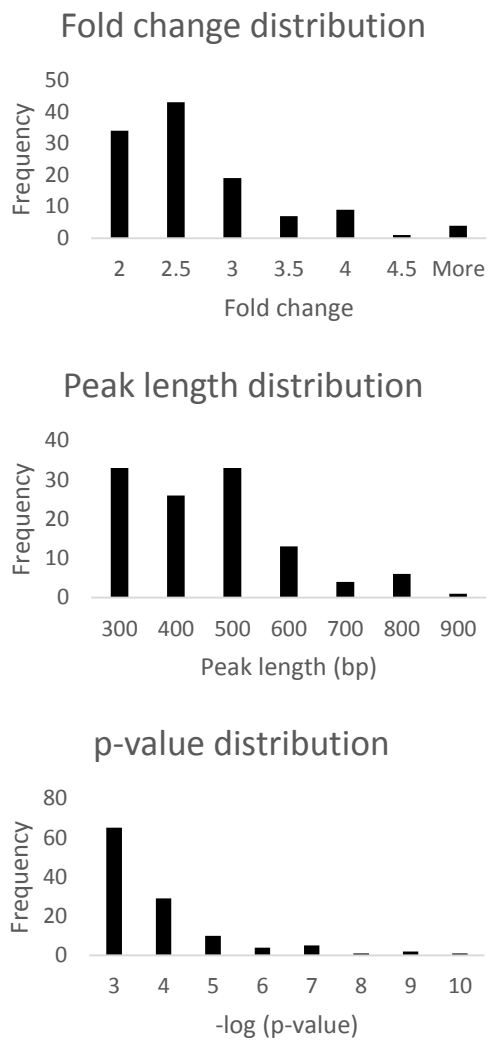

C

### Genomic Background

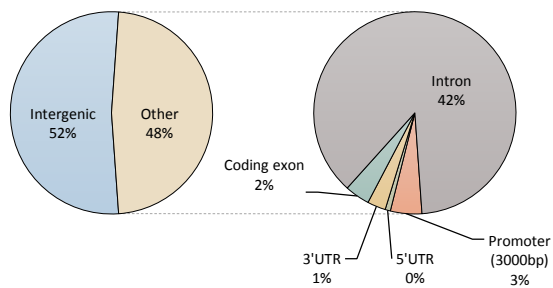

D

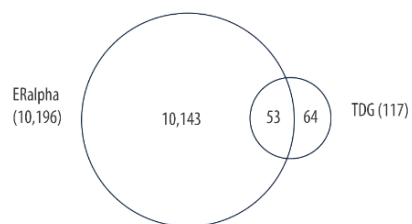

Supplement: Supplementary file 3 — Additional file 3. ChIP-Seq treatment and processing. (A) Reads were mapped to the human genome (hg19) and peaks were built using Partek Genomic Suite. Peaks which occurred in both replicates were filtered based on p value (0.05) and fold change (1.2 fold) to build the final set of 117 high-stringency peaks. (B) Distribution of fold changes (Treatment/Control), peak lengths and p values. (C) Genomic features of hg19 genome build (D) Venn diagram showing overlap between E2-dependent TDG peaks and ER peaks obtained from public dataset. [file 13072_2018_176_MOESM3_ESM.pdf]

| Rank | Motif                                                                              | Name                        | P-value  | % of Targets Sequences with Motif |
|------|------------------------------------------------------------------------------------|-----------------------------|----------|-----------------------------------|
| 1    | 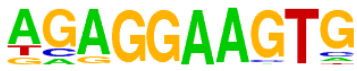 | PU.1(ETS)/Thio<br>Mac-PU.1- | 1.00E-02 | 11.43%                            |

Supplement: Supplementary file 6 — Additional file 6. Motif analysis performed on sites of E2 mediated TDG localization that do not overlap with ER binding, revealed enrichment only for single motif. [file 13072_2018_176_MOESM6_ESM.pdf]

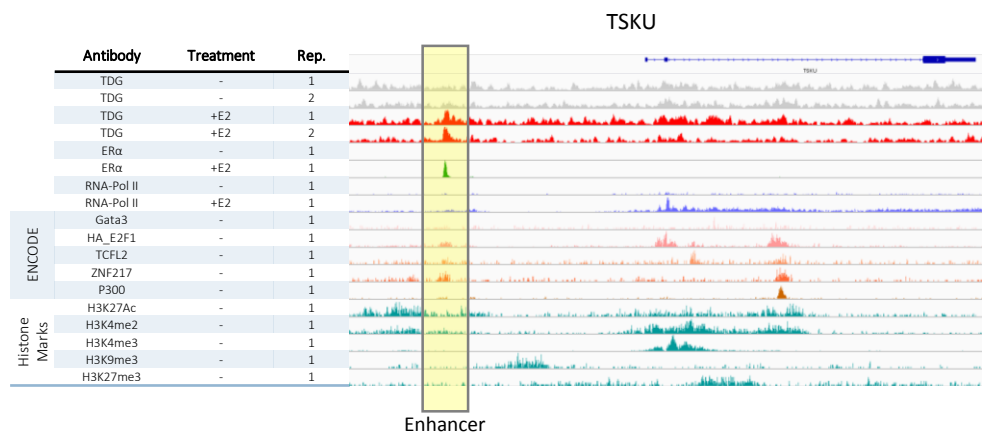

Supplement: Supplementary file 8 — Additional file 8. Genomic landscape at TSKU. Genomic region at E2-dependent TDG binding site of TSKU, a E2-mediated target gene. Shown are TDG, E2, ENCODE subset of transcription factors and histone marks. [file 13072_2018_176_MOESM8_ESM.pdf]

A

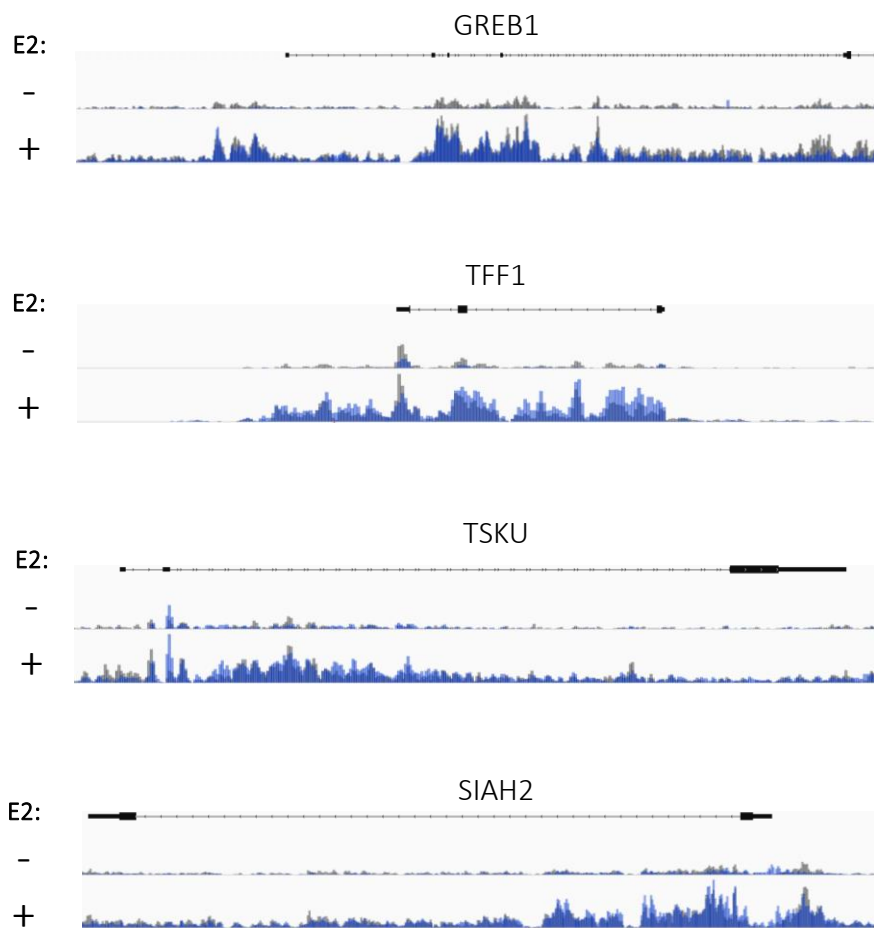

B

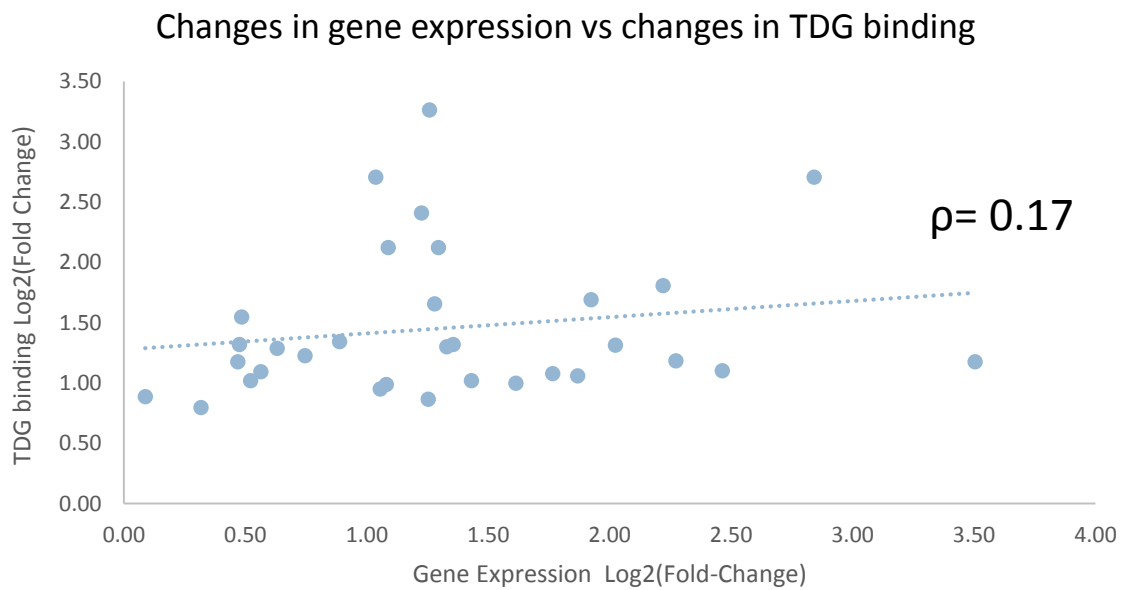

Supplement: Supplementary file 9 — Additional file 9. E2 dependent transcription of genes bound by TDG (A) Transcription levels in response to E2 at genes which bind both TDG and ER. (B) Relationship between TDG binding intensity and gene expression reveals a weak relationship between how much TDG is recruited and gene expression. [file 13072_2018_176_MOESM9_ESM.pdf]

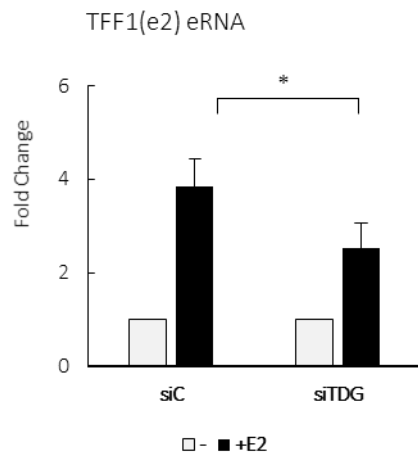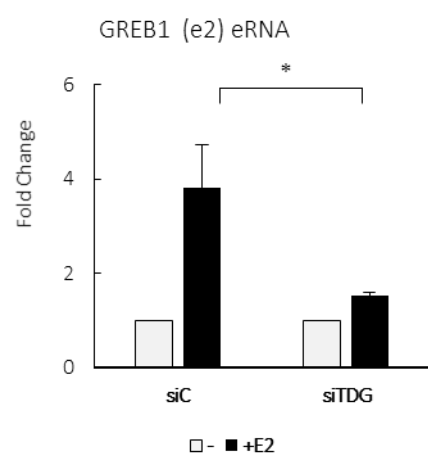

Supplement: Supplementary file 10 — Additional file 10. eRNA production at GREB1 and TFF1. MCF7 cells were depleted of TDG using siRNA, and transcript levels were measured in response to 100 nM of E2 (1 h) (qPCR, p value < 0.05). [file 13072_2018_176_MOESM10_ESM.pdf]

### MAB-Seq at GREB1 enhancer

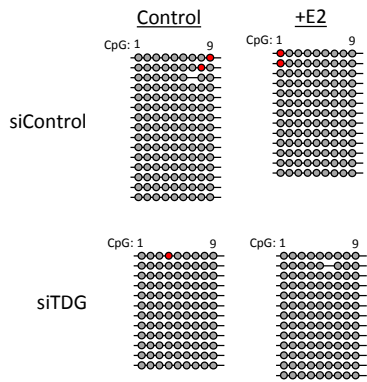

Supplement: Supplementary file 11 — Additional file 11. MAB-Seq of GREB1 enhancer region. Cytosines in the GREB1 enhancer region which are mostly unmodified, regardless of TDG status or E2 treatment. [file 13072_2018_176_MOESM11_ESM.pdf]

A

TDG (chr12:104,357,593-104,384,656)

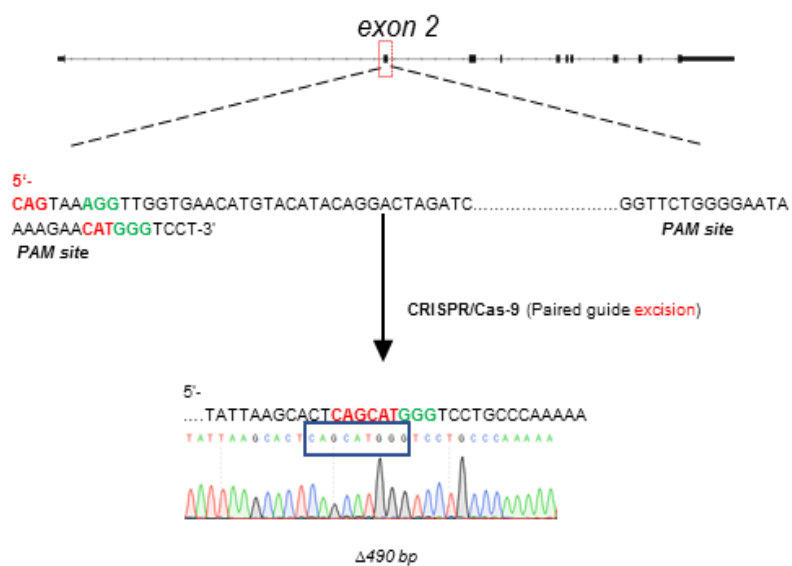

B

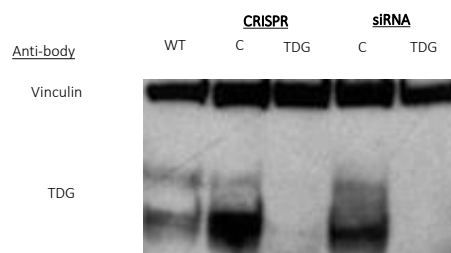

Supplement: Supplementary file 13 — Additional file 13. CRISPR-mediated deletion of TDG in MCF7 cells. (A) To eliminate TDG protein from MCF7 cells we used the CRISPR/Cas-9 and pair guided excision to remove a 490 bp region of TDG which contained exon 2 and which also created a frame-shift. (B) Protein levels of TDG in wild-type (WT) cells as well as those edited using CRISPR or siRNA (C: control siRNA or nontargeting CRISPR, TDG: siRNA targeting TDG or CRISPR targeting TDG). [file 13072_2018_176_MOESM13_ESM.pdf]
